# Of maps and grids

**DOI:** 10.1093/nc/niab022

**Published:** 2021-09-21

**Authors:** Matteo Grasso, Andrew M Haun, Giulio Tononi

**Affiliations:** Department of Psychiatry, University of Wisconsin-Madison, Madison, WI, USA; Department of Psychiatry, University of Wisconsin-Madison, Madison, WI, USA; Department of Psychiatry, University of Wisconsin-Madison, Madison, WI, USA

**Keywords:** integrated information theory, consciousness, contents of consciousness, functionalism, space

## Abstract

Neuroscience has made remarkable advances in accounting for how the brain performs its various functions. Consciousness, too, is usually approached in functional terms: the goal is to understand how the brain represents information, accesses that information, and acts on it. While useful for prediction, this functional, information-processing approach leaves out the subjective structure of experience: it does not account for how experience feels. Here, we consider a simple model of how a “grid-like” network meant to resemble posterior cortical areas can represent spatial information and act on it to perform a simple “fixation” function. Using standard neuroscience tools, we show how the model represents topographically the retinal position of a stimulus and triggers eye muscles to fixate or follow it. Encoding, decoding, and tuning functions of model units illustrate the working of the model in a way that fully explains what the model does. However, these functional properties have nothing to say about the fact that a human fixating a stimulus would also “see” it—experience it at a location in space. Using the tools of Integrated Information Theory, we then show how the subjective properties of experienced space—its extendedness—can be accounted for in objective, neuroscientific terms by the “cause-effect structure” specified by the grid-like cortical area. By contrast, a “map-like” network without lateral connections, meant to resemble a pretectal circuit, is functionally equivalent to the grid-like system with respect to representation, action, and fixation but cannot account for the phenomenal properties of space.

**Highlights**

- Neuroscientific methods successfully account for a system’s functional properties in objective, physical terms but leave out the subjective properties of the accompanying experience.
- We argue that phenomenology can be studied scientifically and in objective terms by unfolding the “cause-effect structure” of physical powers specified by a system.
- When fixating on a target, we experience visual space as extended. Here, we implement a fixation function in two systems—a map and a grid—and we unfold their cause-effect structure.
- We demonstrate that a map can be functionally equivalent to a grid in performing fixation, but only the grid can specify a cause-effect structure that accounts for the extendedness of phenomenal space.

## Introduction

Imagine looking straight at a dark screen. A bright dot appears to the right, and you turn your eyes so that now it appears at the center of the visual field. When we study the brain as neuroscientists, we would like to fully explain how the position of the dot is represented in the brain, how the brain acts to move the eyes such that the bright dot is brought to the center of the retina, and, more generally, how the brain can perform this “fixation” function. This is in fact such a basic function that we have by now a good understanding of how many of its components are implemented. We know how the position of visual stimuli is represented in various brain areas across the occipital and parietal cortices (Wandell and Winawer 2011); we know that the frontal eye fields and other neural circuits control the eyes and that these circuits together can accomplish fixation and pursuit (Krauzlis *et al.* 2017). And we can describe what happens in terms of encoding, decoding, “information processing” (Kriegeskorte 2015), and, when appropriate, “predictive processing” (Hohwy 2013; Friston 2019).

But we also know that when we look at the bright spot on the dark screen, we actually “see” it, and we see it located first at the right and then in the center of visual space. This part we do not understand. How is it that the representation of the position of the dot over the screen, the action of the eyes, and the fixation function they implement to change the representation are accompanied by us “seeing” the dot in space? This part is not function but phenomenology (subjectivity, experience, consciousness, or words to that effect), and unlike function, we do not know how to account for phenomenology in objective, physical terms, which is what neuroscience deals with.

In this paper, we argue that it is possible to provide an objective, neuroscientific account of what it takes for phenomenology to accompany function; in this case, the phenomenal properties of spatial experiences accompanying a fixation function. What needs to be done is to provide a neuroscientific account of what spatial experience is like, not just of what we do with it. We also show that it is possible to dissociate function from phenomenology. A brain constituted of maps connected to each other to perform sensory-motor transformations can perform a function such as fixation without being accompanied by phenomenology. A brain constituted of grids rather than maps, simply by the addition of lateral connections, is functionally equivalent to a map brain in its ability to fixate and follow a target just as well. However, unlike a map brain, a grid brain can account for the key phenomenal properties that compose the structure of spatial experiences. We must be grids, not maps.^1^

## Methods

### Networks

**Figure 1.**
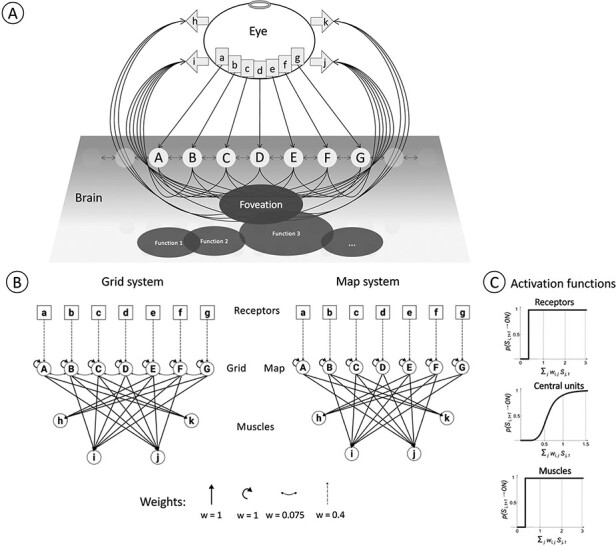
The fixation network. (A) The receptor layer (the rectangles) is enclosed in an eye with a narrow aperture. The receptors send outputs to the brain, which sends outputs to muscles that move the eye. The brain will have other regions that carry out other functions, but, here, we are interested only in the spatial “fixation” function that is carried out by the three layers shown explicitly in (B). (B) Grid and map versions of the network. The input layer of each network is the same: an array of receptors (a–g) that send their sole output to a unit in the middle layer. The middle layer of both systems (the “brain”) consists of seven self-connected units (A–G), each of which receives a single external input from one receptor. Each of these units sends outputs to several muscle units (h–k) in the top layer. No layer of the network sends outputs to any earlier layer. The grid and map are distinguished by the presence of lateral connections in the grid. (C) Activation functions for each unit type.

Our study is focused on two similar systems, one built around a grid and the other around a map (Fig. 1A). Both grid and map systems are constituted of probabilistic binary-state units in three layers: a receptor layer of seven units (the “retina,” *abcdefg*) that feed into the brain, a middle layer of seven self-connected units (the “brain,” *ABCDEFG*), and a motor layer of four motor units that get inputs from the brain (the “muscles,” *hijk*). The two systems differ only in the internal connectivity of the middle layer. In the first system, the middle layer is a grid: each of its units gets input from itself and from a single receptor, and is connected to other units in the same layer by weak nearest-neighbor lateral connections. In the other system, the middle layer is a map: each of its units gets input from itself and from a single receptor, effectively mapping the receptor array but with no further within-layer interactions.

The brains of these simple systems are meant to be reminiscent—of course in a merely schematic manner—of different parts of the visual brain that play important roles in the representation of visual space and the control of eye movements. The grid is like visual cortical areas, which conserve retinal topology but also have dense intra-areal connectivity, including lateral connections between similar cell types (Stettler *et al.* 2002). The map is more like the pretectal nuclei, such as the nucleus of the optic tract, which also conserve retinal topology but have little or no connectivity between their principal neurons (Gamlin 2006). The wiring of the two systems is shown in Fig. 1B.^2^

Each grid or map unit has a strong self-connection weight of *w* = 1, and each has a weaker input from its retinal receptor with weight *w* = 0.4. Grid units have inputs from their nearest linear neighbor or neighbors with weight *w* = 0.075. A grid or map unit passes the sum of its inputs through a compressive nonlinearity (Albrecht and Hamilton 1982), which gives the probability of the unit being ON in the next time step (*t* + 1):

(1) \begin{equation*}P\left( {{S_{i,t + 1}} \to ON} \right) = {{{{\left( {\mathop \sum \nolimits_j {w_{i,j}}{S_{j,t}}} \right)}^n}} \over {{z^n} + {{\left( {\mathop \sum \nolimits_j {w_{i,j}}{S_{j,t}}} \right)}^n}}}\end{equation*}

(2) \begin{equation*}P\left( {{S_{i,t + 1}} \to OFF} \right) = 1 - p\left( {{S_{i,t + 1}} \to ON} \right)\end{equation*}

The parameter *z* = 0.63 is the input level at which the unit has a probability of 0.5 to be ON in the next time step; *n* = 5 sets the steepness of the nonlinearity. The muscle units are noiseless OR gates, turning ON if any of their input units is ON. The retina units are perfect receptors: a retina unit always activates when intersecting a stimulus ray and is inactive otherwise (the activation of each type of unit is shown in Fig. 1C).

### Implementation

We implemented the grid and map systems in an action-perception loop, where the muscles can change the position of the retina with respect to an external stimulus. The receptors are enclosed in an eye with a small aperture directed toward a stimulus domain (Fig. 1A). Each receptor is a bin with width = 1, and each abuts its neighbors with no space between. The muscles pull leftward or rightward, moving the eye and changing the direction of the aperture. When a muscle is activated, the eye is not immediately pulled with full strength; instead, the full muscle strength is approached exponentially, and the eye moves gradually. The instantaneous velocity of the eye in the next time step is *V_t_*_+Δ_*_t_*, which depends on the eye’s velocity at time *t*, the maximum velocity *mM_t_*, and the time constant τ.

(3) \begin{equation*}{V_{t + \Delta t}} = {V_t} + {{m{M_t} - {V_t}} \over \tau }\end{equation*}

*M_t_* is the sum of muscle states at time *t* (where an active Left muscle adds −1, an active Right muscle adds +1, and inactive muscles add 0). The parameter *m* determines how fast the eye can move (*mM* is the asymptotic velocity for muscle state *M*) and was set at *m* = 0.075; the time constant τ controls how long it takes the eye to reach the asymptotic velocity and was set at τ = 10. The eye’s velocity at time *t* determines the change in position *S* over the unit time step (Δ*t* = 1):

(4) \begin{equation*}{S_{t + \Delta t}} = {S_t} - {V_t}*\Delta t\end{equation*}

## Function

When we study a system as neuroscientists, we are interested in studying how systems represent stimuli coming from the environment and how they act based on their inner workings. Here, we employ several approaches typical of neuroscience. We analyze how systems represent and encode information about the input (and how such information can be decoded from internal states of the system), how they act, and, more generally, the functions they perform.

**Figure 2.**
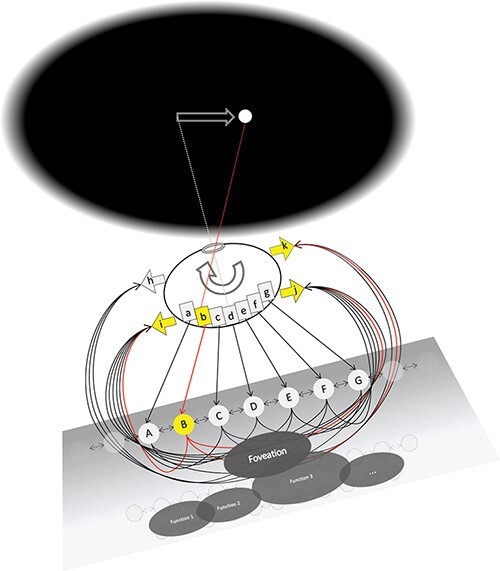
Foveation function. The grid/map system is foveating a dark spot on a dark screen. To the right of the foveated spot, a bright dot stimulates one off-center receptor (“b”) in the eye. This produces an imbalance of motor activity that will rotate the eye to the right so that it will come to foveate the bright dot. Active connections are drawn in red, and inactive connections in black.

In Fig. 2, the system (it does not matter whether grid or map) fixates a blank region on a video display: a bright dot appears off to one side and the system moves its eye to fixate the dot. As this overt behavior plays out, the system’s brain goes through a series of state changes that are measurable using various neuroscientific tools. Studying the system’s behavior or its neural states can address two aspects of the system: what it *does* and what it *represents*.

### What the systems do

When the stimulus domain is “dark” and the grid or map is inactive, the muscles are also inactive, and the eye does not move. When a stimulus (a “bright dot,” for example) is presented to the system and falls on a receptor, that receptor is immediately activated. It typically takes a few time steps for a grid/map unit to be activated by an active input. When it is active, a grid/map unit activates its target muscles. If the grid activity is only to one side of the central unit D (i.e. only in units ABC or only in units EFG), the muscle state is imbalanced, and the eye is pulled in one direction more than in the other so that it shifts toward the stimulus. If the grid activity is balanced around the central unit (or if only the central unit is active), then the muscle state is balanced, and the eye does not move: the eye has fixated the stimulus.

**Figure 3.**
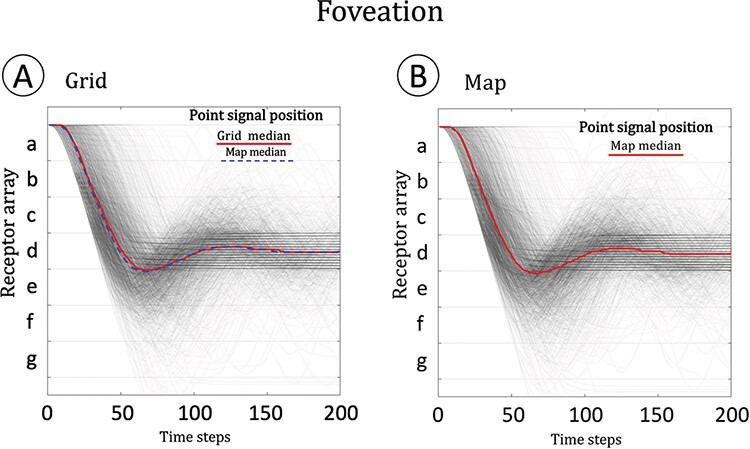
Fixation in the grid and map networks. A target stimulus was placed at the outer edge of the receptor array, just in view of receptor “a.” Each receptor is a bin, the space between the horizontal grid lines. Each gray line traces an individual simulation of the eye’s response to the target: the stimulus position never changes, but the eye moves to fixate it, meaning that the stimulus comes to fall on receptor “d.” The red lines indicate the median of each system over 2000 simulations. The grid and the map behave very similarly—on the left, the grid and map median performance is shown to be indistinguishable.

Figure 3 compares the fixation behavior of the grid and map systems. A stationary point stimulus is placed at the far edge of the eye’s field of view (just on the outside edge of receptor “a”). Each system moves so as to center the stimulus on receptor “d.” Both systems typically fixate the target within 50 time steps, and both are comparably variable in their performance (the variability coming from the noisy mechanisms of the grid/map). The eye moves gradually because just as it takes time for the receptors to evoke activity in the grid/map (due to the low strength of the input connection), it also takes time for grid/map units to become inactive once their receptor inputs are silent (due to the strong self-connections of the grid/map units). If the eye moved more quickly, it would be driven excessively by internal activity traces, would overshoot targets, and would rarely achieve fixation. Nevertheless, both grid and map system are nimble enough to follow a moving target as long as its speed averages less than a receptor’s width per time step.

### What the systems represent

**Figure 4.**
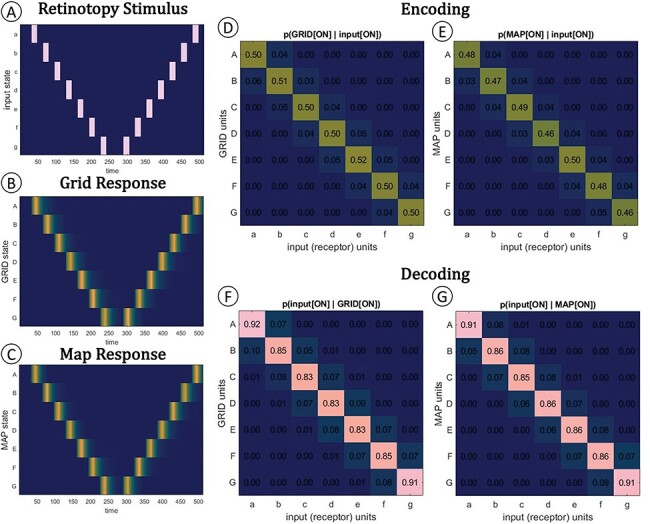
Retinotopy, encoding, and decoding in the grid and map systems. Both the grid and map networks are retinotopic maps of the input layer. (A) The retinotopy stimulus was a bright dot shown to each receptor for 16 time steps, followed by 16 steps of blank display, followed by a stimulus at the next position, etc. When the stimulus had crossed from one end of the retina to the other, it repeated in the opposite direction. The whole 512 time step stimulus was repeated 16 times for each of 20 identical grid or map networks, roughly emulating the parameters of an fMRI retinotopy experiment (with its TRs, trials and repeats, and many subjects). Throughout this experiment, the eye’s muscles were disabled. (B) Grid and (C) map respond similarly to the stimulus. Colors reflect the average proportion of activations over the 320 trials. (D) and (E) show how the stimulus is encoded into grid or map: if an input unit is ON, its target in the grid or map has about *P* = 0.5 of being ON in the next time step. (F) and (G) show how the input can be decoded from grid or map responses: if a grid or map unit is ON, then there is about *P* = 0.85 that its input unit was ON in the previous time step.

In the human brain, aspects of visual space are represented in the spatial organization of neurons in visual cortex. The receptive fields of cortical neurons tile the area of the retina so that the surface of the visual cortex preserves the ordering of points on the retina: this is known as retinotopy (Engel *et al.* 1997). Retinotopy can be demonstrated by stimulating different points on the retina and observing which neurons are activated by the stimulus. We can do the same with our system, stimulating each receptor and examining what happens to each grid or map unit. Figure 4A shows the results of such an experiment: we simulated an fMRI-style retinotopy experiment, in which a point stimulus slowly drifts across the system’s field of view. Both systems respond similarly (Fig. 4B and C). For both systems, the topographic mapping is clear: each receptor in the retina activates one unit in the grid or map. Apart from the topographic mapping, both systems are very similar both in how they encode stimulus position (Fig. 4D and E) and in how they can be used to decode stimulus position (Fig. 4F and G). By any definition, both systems represent the space of the model retina. If we assume that the retina is exposed to an outside world with enduring spatial structure, both systems can be construed as representing that world’s spatial structure.

### How the systems function

**Figure 5.**
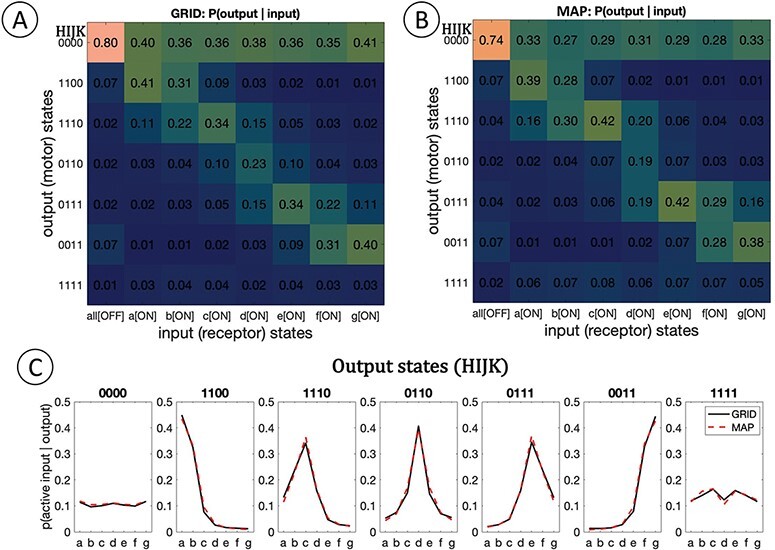
Input/output (i/o) functional descriptions. Each system was run through 10 000 iterations of the “drifting target” condition for 200 time steps each. This i/o description emulates a “perturb each input unit and observe average system response” experiment. Each matrix (A, B) shows the probability of some output state (of the muscle units, listed on the y-axis) given some single-unit-ON input state (of the receptor units, on the x-axis). Not all output states are possible for either system (e.g. HIJK = [0101] is not possible); only possible states are listed. (C) “Tuning functions” on the input array, given each possible output state. Five of the seven possible output states are associated with a selective tuning function on the input. Grid (black lines) and map (red dashed lines) systems hardly differ in this property.

We can also assess the systems in terms of their input/output (i/o) functions (Doerig *et al.* 2019). In our case, this means characterizing the system’s output (muscle) states as a function of a range of input (receptor) states. Figure 5 shows the i/o characteristic of the grid and map systems as the probability of each possible muscle (muscle-layer) state given a particular one-receptor-ON input state. The probabilities were obtained by running 10 000 iterations of a “moving target” simulation, in which the stimulus point wanders randomly across the stimulus domain, for 200 time steps each.

The i/o characteristics of grid and map are extremely similar, qualitatively and quantitatively (the functions are correlated with ρ = 0.9715). This is due to the fact that they map their inputs in a similar way (Fig. 4) and to the fact that the grid and the map project in exactly the same way to the muscle layer (Fig. 1). The i/o characteristic of each system can also be inverted so that each output state is associated with a certain “tuning function” on the input domain (i.e. the probability of input unit activity given some output state; Fig. 5C). The tuning functions are virtually identical for both systems. Only five of the seven possible tuning functions are selective. Judging from the system’s i/o characteristic, one might conclude that this system only represents five overlapping spatial positions.

Minor differences in the i/o functions between the two systems are due to a “modulatory” role of lateral connections in the grid system. Due to the facilitatory influence of lateral connections, grid units are activated by retinal inputs a little faster than map units. Conversely, grid units are slower to deactivate once their input has deactivated, since they may now have an ON neighbor that sustains their activity. Nevertheless, the grid and map system fixate with roughly the same speed and accuracy (For the purpose of this work, the map and grid systems have been built to be functionally equivalent. However, as pointed out by one anonymous reviewer, the subcortical and cortical pathways mentioned above likely have different functional properties reflecting a trade-off between propagation/reaction speed and information integration). Altogether, the two systems are functionally equivalent: they represent stimuli, act on them, and perform the same i/o function, ultimately fixating and following targets in very similar ways.^3^

## Phenomenology

### The phenomenal structure of space

The standard tools of neuroscience let us fully account for the functioning of the grid and map systems in objective, physical terms. At the end of the analysis, we fully understand how both systems work—how they represent retinotopic spatial positions, how they bring about appropriate eye movements, how their units are tuned, and how the two systems can perform the fixation function. The human brain, of course, is immensely more complicated. However, an account along the lines of the one just provided would seem adequate for explaining how our visual system can perform a function such as fixation. A larger number of neurons and areas will be involved, but the principles do not change.

However, such an account leaves completely unexplained how, as human subjects, we not only have the ability to fixate the bright dot but also experience it as a particular region of space at its particular location, first at the periphery and then at the center of an extended visual field. Can we provide a neuroscientific, objective account of the phenomenal, subjective properties of our experience of space—of the extendedness of the visual field, with all its regions and locations?

Integrated Information Theory (IIT; Oizumi *et al.* 2014; Tononi *et al.* 2016) has the explicit goal of accounting for the phenomenal properties of experience in physical, causal terms. The initial focus has been on the essential properties of consciousness—those that are true of every conceivable experience. More recently, the tools of IIT have been employed to address the properties of specific experiences, beginning with those of spatial experience (Haun and Tononi 2019).

**Figure 6.**
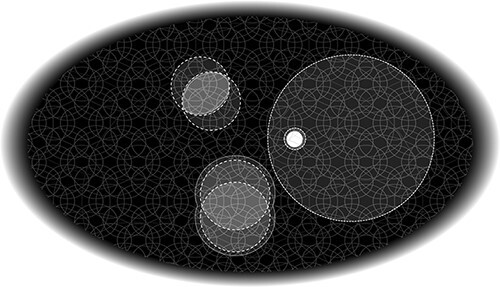
Phenomenal space. The phenomenal experience of visual space is of a canvas filled with spots (distinctions) of any size, related among themselves in a specific “spatial” way: every spot overlaps itself and overlaps with other spots according to connection, fusion, and inclusion (see text and Haun and Tononi (2019) for further explanation).

Briefly, IIT begins by considering the fundamental phenomenal properties of spatial experiences—those that make space feel like an extended canvas (Fig. 6). Introspection and reasoning indicate that the phenomenal structure of space is composed of a large number of phenomenal distinctions or “spots” bound by an even larger number of relations among them. Specifically, any spot overlaps itself (reflexivity); for any given spot, we can always find a spot partially overlapping it such that their intersection is also a spot (connection); we can find a spot partially overlapping it such that their union is also a spot (fusion); and we can find a spot that includes it or is included by it (inclusion) (Haun and Tononi 2019). These four phenomenal properties are what makes the experience of space feel “extended.” Other properties of spatial experience, such as regions, locations, sizes, boundaries, and distances can be derived from these fundamental properties. The next step is to account for these subjective, phenomenal properties in objective, neuroscientific terms, just as we do for functional properties.^4^

### Cause-effect structures: unfolding the causal powers of a system

According to IIT, to achieve this goal, we need to establish not just what a physical substrate can *do*—how it performs its functions—but what it “is”—what its full causal powers are at a given moment. The tools of IIT can be employed to fully unfold the causal powers of a substrate such as a network of neurons, some active and some not, by systematically manipulating and observing each subset of the network’s units. Doing so yields its “cause-effect structure,” which expresses the irreducible causes and effects of every mechanism of the system (causal “distinctions”) as well as the causes and effects they specify jointly (causal “relations”). To qualify as a physical substrate of consciousness, the cause-effect structure specified by a neural system must satisfy the essential phenomenal properties of experience in physical terms. Its cause-effect power must be intrinsic (within the system), structured (composed of causal distinctions and relations), specific (specifying particular cause and effect states), unitary (irreducible to causally independent subsystems), and definite (having a border and grain). If these essential properties are satisfied, the particular cause-effect structure specified by the system in its current state should account in full for the particular phenomenal structure of its current experience (Tononi 2015).^5^

**Figure 7.**
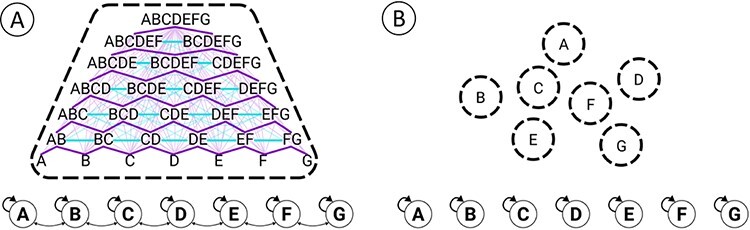
Cause-effect structures of grid and map. (A) The grid specifies a single cause-effect structure (indicated by the single dashed line) composed of 79 causal distinctions (“spots”) and a large number of relations among them. Here, only a representative subset of the distinctions and relations composing the structure is shown. Cyan edges indicate that two distinctions are related by connection and fusion; purple edges indicate that one distinction includes the other. The structure suggested here is that specified by the “all OFF” grid. The state of the system does affect the existence of the grid’s distinctions (Haun and Tononi 2019). (B) The map specifies seven independent, minimal cause-effect structures (indicated by the seven dashed outlines) each composed of a single first-order distinction.

In the case of the grid system, the unfolding procedure reveals that the middle layer—the grid itself—satisfies the essential requirements for supporting experience as a single entity.^6^ In any system state, the grid specifies a single cause-effect structure (indicated by the dashed outline of the cause-effect structure in Fig. 7A) composed of 79 causal distinctions and a large number of causal relations, a few of which are suggested in Fig. 7A.

For example, there are seven first-order distinctions, specified by each individual unit with a cause and an effect over itself (due to the self-connection). There are 11 second-order distinctions, specified by pairs of nearby units over themselves or their mutual neighbors (e.g. AB, AC, BC, BD, CD, … FG). These distinctions are irreducible because, due to lateral connections, pairs of nearby units share inputs and outputs. By contrast, pairs of distant units (say, AD, AE, … AG) do not share inputs and outputs and their causes and effects are reducible to independent subsets. The remaining higher-order distinctions, all the way up to ABCDEFG, obey a similar pattern. Causal relations correspond to the irreducible overlaps among causes and effects specified by the 79 distinctions.

Unlike the grid, the map is not a single, irreducible entity. The unfolding procedure reveals that, due to the lack of lateral connections, the map breaks down into seven causally independent units, each specifying a single, first-order distinction over itself (Fig. 7B, indicated by the seven dashed outlines). From an extrinsic, functional perspective, the grid and map systems may appear fundamentally similar—they represent, act, and function similarly. But from an intrinsic, causal perspective, they could not be more different. The grid, it turns out, is a single, irreducible entity that specifies a rich cause-effect structure; the map, by contrast, is not an entity but an aggregate that reduces to seven mini-entities, each specifying a minimal cause-effect structure.

### The correspondence of phenomenal and cause-effect structures

According to IIT, the particular phenomenal properties of an experience supported by a physical substrate in a particular state should correspond one-to-one to the particular cause-effect structure it specifies (Oizumi *et al.* 2014; Tononi *et al.* 2016). As previously shown (Haun and Tononi 2019), the cause-effect structure specified by a grid-like system can in fact account for the specific properties that characterize the experience of spatial extendedness—the fundamental properties of reflexivity, connection, fusion, and inclusion and derived properties such as regions, locations, and distances.

**Figure 8.**
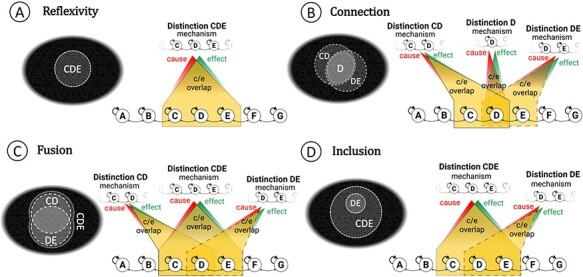
Extendedness. Phenomenal spots are represented in the top left of every panel; the corresponding distinctions are represented in the rest of the panel. For simplicity, spots are represented as 2D, although the space specified by a 1D grid would be 1D. (A) Phenomenal spatial distinctions (“spots”) are reflexive: they overlap or “point to” themselves. A causal distinction from the grid (here, the system is in the all OFF state) also overlaps itself, yielding a reflexive relation. (B) Spots connect to one another: when they overlap partially, their intersection is also a spot. When causal distinctions from a grid overlap partially, another distinction exists corresponding to the intersection. (C) Spots fuse with one another: when they overlap, their union is also a spot. When causal distinctions from a grid overlap, another distinction exists corresponding to their fusion. (D) Spots include one another in the sense that one spot might fully overlap another as well as some distinct spot. Distinctions from a grid often overlap in a corresponding way. The overlapping spots inset in each panel are labeled with the analogous distinction in the example, but it should be clear that the space of the line grid is 1D, not 2D like the phenomenal space of Fig. 6.

For the cause-effect structure specified by the grid system, some of these properties are represented schematically in Fig. 7A. The correspondence between the subjective properties that characterize phenomenal extendedness and the objective properties that characterize extendedness in physical, causal terms is shown in Fig. 8. Every causal distinction overlaps with itself in the sense that its cause overlaps with its effect (reflexivity, Fig. 8A); for every distinction, one can always find another distinction that partially overlaps it such that their intersection is also a distinction (connection, Fig. 8B); a distinction that partially overlaps it such that their union is also a distinction (fusion, Fig. 8C); and a distinction that includes it or is included by it.^7^ None of this applies to the map system given that, due to a lack of lateral connections, it does not even specify a single cause-effect structure. To support extendedness, connections must be organized in a grid-like manner. For example, a network with as many units and connections as the grid system, but organized at random, fails to satisfy reflexivity, connection, fusion, and inclusion (Haun and Tononi 2019).

Of course, the cause-effect structure specified by a 1D, 7-unit grid is minimal, compared to what we might expect to be the cause-effect structure specified by the stack of 2D cortical areas in the human visual cortex, constituted of hundreds of millions of neurons. Nevertheless, an account along the lines of the one just provided would seem adequate, in principle rather than in degree, to explain the fundamental structural properties of phenomenal space (see also Haun and Tononi (2019) for a 2D example).

## Conclusions

The simple networks analyzed here represent the position of a stimulus and act on its representation to fixate it. The systems’ functions can be fully explained by examining the tuning functions of their units, how they encode stimulus positions (which can be decoded from their activity patterns), and how they activate ocular muscles. This holds whether a system is as simple as these networks or as complex as the human brain and whether it can merely follow and fixate a stimulus or recognize and report the identity of a face in a crowd. Many assume that a full functional explanation of what a system does, and how it does it, exhausts what we should and can hope to understand in objective, neuroscientific terms [we discuss this point in detail in a companion paper (Ellia *et al.* 2021)].

However, even when we merely fixate a stimulus, regardless of any functional response, we also “see” it, and we see it located in space, first at the periphery and then at the center of the visual field.^8^ To account for the fact that space feels extended and that stimuli appear at their experienced location within space, we cannot just rely on a functional account. Instead, we need to account for the phenomenal structure of spatial experience. As illustrated here, phenomenal properties can be accounted for in objective, neuroscientific terms just as much as functional properties. To achieve this goal, however, we must assess what a system “is”—its causal powers at a given moment—rather than what a system “does”.

Drawing on IIT (Oizumi *et al.* 2014; Tononi *et al.* 2016) and its application to the problem of spatial experience (Haun and Tononi 2019), we have shown how the causal powers of a substrate can be characterized in full by unfolding its cause-effect structure. Doing so reveals that a grid-like substrate constituted of units linked by lateral connections specifies a cause-effect structure whose causal distinctions and relations can account for the phenomenal distinctions and relations that characterize the experience of space. The fundamental phenomenal relations of reflexivity, connection, fusion, and inclusion among spots—and the derived properties of regions, locations, sizes, boundaries, and distances, which compose the phenomenal structure of subjective space—find an objective correspondent in the causal properties of cause-effect structures specified by grid-like substrates.^9^

We have also shown that a map-like substrate—one that lacks lateral connections—can fixate just as well as a grid-like substrate—it is functionally equivalent to it (or nearly so). However, unfolding a map does not even yield a single cause-effect structure, let alone one that is spatially organized. It follows that while the map network is functionally equivalent to the grid network, it is not phenomenally equivalent to it—there “cannot be” any phenomenology for a map, let alone spatial phenomenology.

An intriguing prediction that follows directly from the dissociation of function and phenomenology concerns the scrambling of input and output connections. For both the grid and map systems described here, if the connections between receptors and central units were scrambled, and those between central units and eye muscles were scrambled in a compensatory manner, there would be hardly any functional consequence. However, for the grid network, the scrambling would result in a major phenomenal change: a dot stimulus moving across the retina would not be experienced first at the far right, then closer and closer to the center, but would appear sequentially at random locations in space.

Elementary as they are, the grid and map networks illustrated here were meant to be reminiscent of certain neural substrates in the human brain. A majority of areas in posterior cortex are organized like stacks of grids. Clinical and experimental evidence from lesion, stimulation, and recording studies indicates that these grid-like cortical areas do in fact support the experience of space (Pollen 1999). For instance, damage to the occipito-parietal lobe of one side results in the loss of a large portion of space not just in perception but also in imagination (Butter *et al.* 1997). In other words, subjects are not just blind to stimuli presented contralaterally to the lesion but are unaware of the very existence of that part of the visual field—in fact, they are often unaware that anything is missing (Townend *et al.* 2007). By contrast, it is generally thought that subcortical visual pathways, such as those running through pretectal nuclei, mediate reflex functions that remain unconscious (Martin 2020). Indeed, damage to these circuits results in various functional deficits (Keane 1990) but has only indirect effects on visual experience. However, it is usually not explained why posterior cortical areas would support the experience of space as well as govern functions such as the smooth pursuit of a visual target, whereas pretectal circuits may implement similar functions (and interact with cortical circuits) without contributing to experience. Perhaps, as proposed here, it may come down to grids versus maps.

## Footnotes

1 To be precise, we must be “unfolded” grids (see below).

2 Two main visuomotor pathways in the human brain (Martin 2020), one cortical and one subcortical, are illustrated in the inset. A cortical pathway starts from the retina and optic nerve, courses through the thalamus (lateral geniculate nucleus), and reaches grid-like areas in occipital visual cortex (VC), parietal areas (PPC), and temporal areas (e.g., hMT), and from there to the frontal eye fields (FEF; cortical areas indicated in blue), which send signals down to the superior colliculus (SC) and other subcortical areas, finally driving the cranial nerves (III, IV, VI) controlling the muscles of the eye. A subcortical pathway, which bypasses thalamus and cortex, goes from the retina directly to pretectal nuclei, map-like nuclei such as the nucleus of the optic tract (NOT, in orange); from there, signals find their way to the same cranial nerves. This depiction of visuo-motor pathways involving “grids” (in the cortex) and “maps” (the pretectum) is of course overly simplified and is only meant to help placing the content of the paper in an anatomical context.

3 The minimalistic model presented here could be augmented with circuits for deploying spatial attention and reporting the position of the bright dot through means other than ocular movements. As argued in the companion paper (Ellia *et al.* 2021), the phenomenal properties of spatial experience must be accounted for independent of whether they are reported or not.

4 For a brief video tutorial about the fundamental phenomenal properties of spatial experience, see https://centerforsleepandconsciousness.psychiatry.wisc.edu/index.php/space-tutorial/.

5 For the mathematical details of computing causal distinctions, see Barbosa *et al.* (2021); for causal relations, see Haun and Tononi (2019). The cause-effect structures of the two systems are unfolded using PyPhi (Mayner *et al.* 2018).

6 The input and output layers are excluded because input units have no causes within the system and output units have no effects within it; for the full unfolding of an 8-unit grid see Haun and Tononi (2019).

7 Inclusion, Fig. 8D; some distinctions represent exceptions to these properties, e.g. the highest-order distinction (*ABCDEFG*)—the “total spot” which is as big as the entire visual space—that is reflexive and includes other distinctions but neither connects, nor fuses, nor is included by others, and the first-order distinctions (*A, B, C, D, E, F,* and *G*)—the “points” of visual space—that are reflexive and included by but neither connect, nor fuse, nor include other distinctions (Haun and Tononi 2019).

8 The focus here is only on accounting for the spatial properties of experience, leaving aside other aspects such as the dot’s brightness, its circular shape, etc. (as discussed in Haun and Tononi (2019)).

9 It is hard to conceive of an alternative explanation for spatial experience in neural terms (see Haun and Tononi (2019)). For example, one might propose that distance is “represented” intrinsically by the number of synaptic steps between neurons in a cortical area. Thus, in the simple case of the grid network, there is one synaptic step between unit A and B, two between A and C, three between A and D, and so on. However, such distances are only explicit in the mind of the beholder, who is already aware of what space is and can sequentially calculate synaptic distances between any two units. In the IIT account, the unfolded cause-effect structure of the grid system offers an explicit physical correspondent to every distance, namely the smallest high-order distinction that overlaps both units. Or take inclusion, say that AB includes A, ABC includes AB, and so on. All inclusion relations among spots have an explicit causal correspondent in the cause-effect structure specified by a grid. Like all distances, all inclusion relations, as well as connection and fusion relations, are simultaneously present in the cause-effect structure. No hierarchical arrangement of neurons with increasing receptive field sizes could explicitly capture all these relations either (Haun and Tononi 2019). And while a neuroscientist (or a computer program) could always perform an appropriate computation (how far is this unit from that, does this receptive filed include that, and so on), it would do so extrinsically and sequentially. The number of phenomenal distinctions and relations that is required to compose an empty canvas and make it feel the way it does is immense and is available intrinsically and simultaneously (Haun and Tononi 2019; Ellia *et al.* 2021).
